# A Cell Density-Dependent Reporter in the *Drosophila* S2 Cells

**DOI:** 10.1038/s41598-019-47652-0

**Published:** 2019-08-14

**Authors:** Matthew L. Romine, Mo Li, Kevin Jiayang Liu, Sapna K. Patel, Julie G. Nelson, Ping Shen, Haini N. Cai

**Affiliations:** 1 Department of Cellular Biology, University of Georgia, Athens, GA 30602 USA; 2 Present Address: Biological and Environmental Science and Engineering Division, 4700 king Abdullah University of Science and Technology, Thuwal, 23955-6900 Saudi Arabia

**Keywords:** Reporter genes, Cell-cycle exit

## Abstract

Cell density regulates many aspects of cell properties and behaviors including metabolism, growth, cytoskeletal structure and locomotion. Importantly, the responses by cultured cells to density signals also uncover key mechanisms that govern animal development and diseases *in vivo*. Here we characterized a density-responsive reporter system in transgenic *Drosophila* S2 cells. We show that the reporter genes are strongly induced in a cell density-dependent and reporter-independent fashion. The rapid and reversible induction occurs at the level of mRNA accumulation. We show that multiple DNA elements within the transgene sequences, including a metal response element from the *metallothionein* gene, contribute to the reporter induction. The reporter induction correlates with changes in multiple cell density and growth regulatory pathways including hypoxia, apoptosis, cell cycle and cytoskeletal organization. Potential applications of such a density-responsive reporter will be discussed.

## Introduction

Cell density sensing and growth regulation are among the most important cellular functions. Signals from developmental programs and cellular environment such as nutrient, oxygen and toxin levels, as well as cell-cell, cell-substrate interactions are integrated to control multiple cellular apparatus to regulate cell cycle, cell fate differentiation, cell migration, cytoskeletal structures, and apoptosis^1–6^. These mechanisms ensure proper proliferation, differentiation, and organogenesis during animal development. Disruption of these functions could alter cell fates and lead to diseases. One of the most important mechanisms that regulate cell proliferation is contact inhibition^2,7,8^. It is known that cell-cell contacts in a crowded culture of normal cells lead to suppression of further cell proliferation, resulting in a confluent cell monolayer. In many cancer cells, such “contact inhibition” is abolished, leading in uncontrolled proliferation. This highlights the *in vivo* relevance of density sensing and responses as critical mechanisms that ensure normal tissue homeostasis. At the center of the highly conserved contact inhibition pathways are a series of phosphorylation events, mediated by the Hippo/Mst1/2/Salvador and the Wts/LATs/Mats kinase complexes, which ultimately lead to the inhibition of the Yorkie/YAP/TAZ transcription factor and the down regulation of genes that promote cell proliferation and survival. Despite recent progresses, the signals and gene regulatory networks that contribute to density sensing and growth control are not fully understood. We report here the development of a cell density reporter system in transgenic *Drosophila* cells^9^. The reporter consists CaSpeR transposon vector and the green or red fluorescent protein (GFP or RFP)^9–17^. In both transiently and stably transfected *Drosophila* cells, these reporters response strongly to changing cell density. We show that the rapid and reversible induction occurs at the level of mRNA accumulation and is mediated by multiple components in the transgene. We present evidence that the transcriptional activation of the reporters is in part mediated by pericellular hypoxia via a *metallothionein* (*MTA*) gene enhancer. However, additional density-dependent signals besides hypoxia are responsible for the strong GFP or RFP induction. The reporter activation correlates with changes in several cellular pathways that respond to cell density. These include markers of cell cycle regulation and apoptosis, the Hippo pathway, a hypoxia marker, and factors involved in cytoskeleton and motility. The density-responsive reporter could be further characterized and customized in its *cis and trans* components to provide cell-based platforms for RNAi or chemical screens for regulators of cell growth and proliferation.

## Results

### Induction of reporter genes by high cell density in transiently transfected Drosophila S2 cells

**Figure 1:**
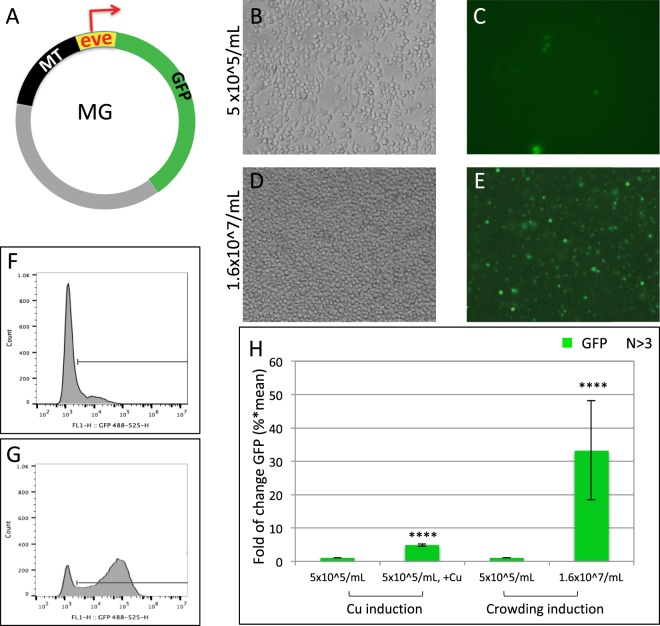
GFP reporter is activated by cell crowding in *Drosophila* S2 cells. (**A**) Schematic of the MT-GFP (MG) transgene. Transgene components are shown in different colors: CaSpeR vector (grey), MT enhancer (black), *evenskipped* basal promoter (light yellow) and the GFP reporter gene (green). The red arrow: Transcription start site (+1). (**B–E**) Differential interference contract (DIC, left) and epifluorescence (right) microscopy images of MG cells at low (5 × 10^5^/mL, **B**,**C**) or high (1.6 × 10^7^/mL, **D**,**E**) culture density. (**F**–**G**) Fluorescence Activated Cell Sorting (FACS) histogram of MG cells at low (5 × 10^5^/mL, **F**) or high (1.6 × 10^7^/mL, **G**) culture density. X-axis: log scale of GFP level; Y-axis: cells number at indicated GFP level. Horizontal bar: GFP positive gate with fluorescence level above 2.5 × 10^3^. (**H**) Quantitation of GFP induction by 1 mM CuSO_4_ and by high cell density. The total GFP fluorescence level is calculated as the percentage of the GFP positive cells multiplied by the mean GFP intensity of these cells. Left, GFP level in MG cells at low density (5 × 10^5^/mL) without CuSO_4_. This level is used as 1 to calculate fold of induction. Middle, fold of GFP induction in low-density MG cells after Cu^++^ induction (see methods). Right, fold of GFP induction in high-density (1.6 × 10^7^/mL) MG cells in the absence of CuSO_4_. N indicates the number of biological replicates. The P-values for the difference between the GFP means of uninduced and induced conditions is marked above the induced data bar.

We have recently discovered that a GFP reporter driven from the MT enhancer of the *Drosophila MTA* gene is strongly induced in *Drosophila* S2 cells at high density (Fig. 1A–E). At 5 × 10^5^/mL, the total GFP level, as defined by Fluorescence Activated Cell Sorting (FACS) assays, is low (Fig. 1B–C,H. see methods). When the culture density increases to 1.4–1.8 × 10^7^/mL, the GFP level raises by over 30 fold, both from an increase in the mean GFP level and the frequency of GFP-positive cells. This occurs even as these freshly transfected cells divide and presumably as the copy number of the transgene reduces (Fig. 1F–H). In comparison, GFP induction by 1 mM Cu^2+^ is only 5–7 folds (Fig. 1H). We found that in S2 cells transfected with an RFP reporter transgene (CA-MT-eve-RFP, MR, Fig. S1A–D), the reporter expression is also strongly induced by high cell density^14^.

### Reporter activation occurs mainly through mRNA accumulation

**Figure 2:**
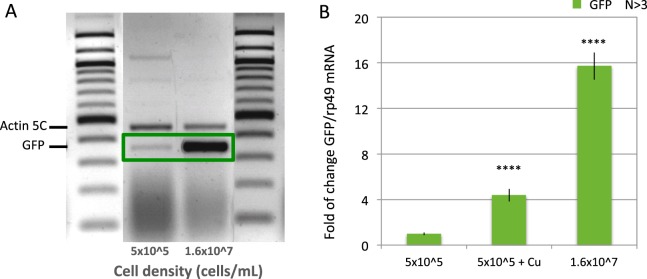
Crowding induction of the GFP reporter occurs at the mRNA level. (**A**) Agarose gel electrophoresis of multiplex RT-PCR product (see methods for details) using GFP and Actin5C primers. Left and right lanes, DNA size marker 100 bp ladder. Center lanes, PCR product from equal number of MG cells of low density (5 × 10^5^/mL, center left) and high density (1.6 × 10^7^/mL, center right). The position of expected Actin 5 C (loading control) and GFP mRNA products are indicted on left. (**B**) Quantitative reverse transcription PCR (RT-qPCR) quantitation of GFP/rp49 mRNA ratio in MG cells at low density (left), MG cells at low density after Cu^++^ induction (left), and MG cells at high density without Cu^++^. N indicates the number of biological replicates. The P-values for the difference between the means of GFP mRNA level in uninduced and Cu- or crowding-induced conditions are marked above the induced data bars.

Gene regulation can occur at many different levels including rate of transcription, mRNA degradation, as well as protein synthesis, modification, maturation and degradation. In order to distinguish whether the reporter activation occurs at mRNA or protein level, we performed reverse transcriptase-mediated PCR (RT-PCR) to assess the reporter mRNA level in low- and high-density cultures, respectively. As seen in Fig. 2A, RT-PCR followed by gel electrophoresis quantitation suggests a ~30x increase in the GFP mRNA level under high cell density over low density, as compared to the Actin 5C control. Quantitative reverse transcription PCR (RT-qPCR) was also used to measure the GFP mRNA and rp49 control mRNA levels. It showed a ~20x induction (Fig. 2B). These induction ratios are comparable to that of GFP protein induction as measured by FACS (Fig. 1H). Comparable protein and mRNA induction is also seen with the RFP reporter (see below). These results suggest that that reporter activation occurs mostly at the level of mRNA accumulation.

### Establishing stably transfected cell lines that exhibit density-responsive reporter activation

A density responsive reporter is useful for biochemical studies and for identifying regulators of growth and proliferation. As such it is imperative to establish and characterize stably integrated transgenic cell lines that provide consistent cell sources and display well-calibrated reporter activation behaviors. P-element-based transposition has been shown to produce predominantly low copy number solitary insertions in stably transfected *Drosophila* cells^18^. We have also reported the use of the *Drosophila* P element transposon for improving efficiency of genomic integration and single copy transgene insertion in the S2 cells^9^. As the first step towards generating stable reporter lines that respond to cell density, we co-transfected S2 cells with the MG or MR transgenes and pTurbo, a plasmid encoding the P element transposase (Fig. S2)^9,19^. The GFP or RFP positive cells were enriched by consecutive FACS sorting over the course of eight months to reach a stable polyclonal cell population. Quantitative PCR indicates that the average transgene copy number in these polyclonal cells is ~10 per cell. Both populations show broad range of expression before and after induction, suggesting large variation in the transgene copy number. The GFP population is less pure and shows a similar crowding induction dynamics as the transiently transfected cells, both in changes in the frequency of positive cells, and in the mean GFP level (Fig. S2A–D, compare to Fig. 1F–H). In contrast, the MR stable population is over 95% positive as judged by its response to Cu^++^. As a result, the MR population shows only a 3-fold increase in crowding induction, reflecting mainly the increase in mean fluorescence (Fig. S2E–H). In summary, both MG and MR transgenes exhibit strong reporter induction by high cell density as integrated copies in the genome and chromosomal environment. These stable transgenic populations are further characterized below to understand the density-mediated induction of reporters.

### Reporter induction is density dependable and reversible

**Figure 3:**
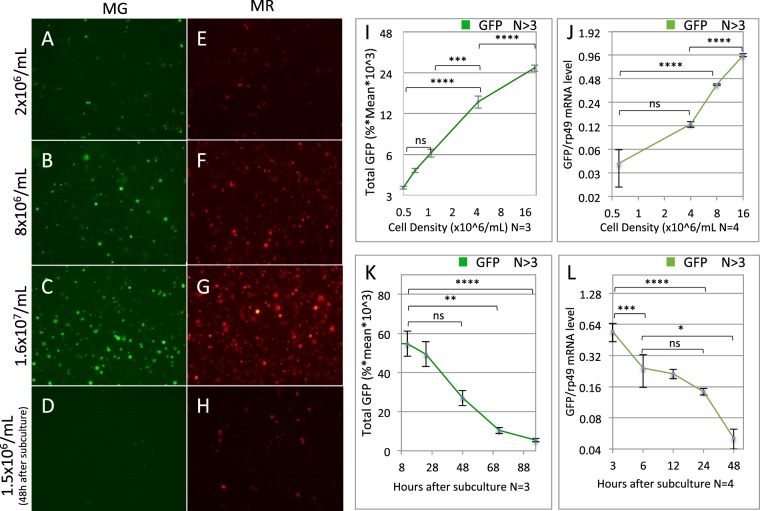
Reporter induction is cell concentration dependent and reversible. (**A**–**H**) Epifluorescence microscopy images of stable MG (**A**–**D**) and MR (**E**–**H**) cells at increasing cell concentration (**A**–**C**,**E**–**G**), and 48 hours after subculture from high cell density (**D**,**H**), respectively. (**I**,**J**) Quantitation of GFP level at increasing cell density, as measured by FACS (**I**), or by GFP/rp49 mRNA ratio using qRT-PCR (**J**). (**K**–**L**) GFP fluorescence (**K**) and mRNA (**L**) levels after increasing length of time following subculture from 1.6 × 10^7^/mL to 5 × 10^5^/mL. N indicates the number of biological replicates. The P-values for pair-wise data comparisons are as indicated by the brackets.

We used the stable MG or MR cells to first examine the dynamics of the reporter induction during cell proliferation. We found that the GFP or RFP level began to rise even at low-intermediate cell concentration (1–2 × 10^6^/mL), when they were still actively proliferating (Fig. 3A,B,E,F,I). The reporter expression continued to increase exponentially with cell density, reaching a peak level at ~1.6 × 10^7^/mL (Fig. 3C,G,I). Similarly, the GFP mRNA exhibited a similar exponential increase with the cell density (Fig. 3J). Importantly, the GFP and RFP induction was reversible when cell density was reduced and maintain at 5 × 10^5^/mL through subculture (Fig. 3D,H,K). The GFP mRNA decreased rapidly, reaching the basal level within 48 hours (Fig. 3L). The GFP protein decayed more slowly, reducing to basal level in 3–4 days (Fig. 3K). This could be due to the perdurance of the fluorescent proteins. These observations are also consistent with the finding that the density-mediated induction occurs mainly at the transcript level.

### Analysis of transgenes for DNA elements that mediate reporter activation

**Figure 4:**
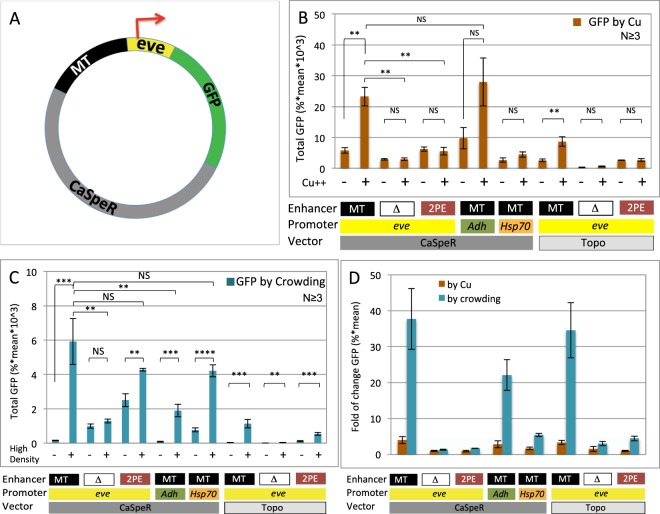
Multiple sequences in the transgene contribute to density-mediated reporter induction. (**A**) Schematic of the MT-GFP (MG) transgene as in Fig. 1A. (**B**) FACS quantitation of GFP induction by Cu^++^ in low density S2 cells containing transgenes with various enhancers, promoters and vectors sequences. The + or − sign indicates the presence or absence of 1 mM CuSO4. (**C**) FACS quantitation of GFP induction by high cell density in S2 cells containing transgenes with various enhancers, promoters and vectors sequences. The + or − sign indicates the high (1.6 × 10^7^/mL) or low (5 × 10^5^/mL) cell density. (**D**) Fold of GFP induction by CuSO_4_ (brown) and cell crowding (blue) in S2 cell containing different transgenes, as indicated. N indicates the number of biological replicates. The P-values for pair-wise data comparisons are as indicated by the brackets.

As the first step towards identifying the *cis* and *trans* components that mediate reporter activation at high cell density, we dissected the transgene DNA elements in reporter induction assays. Besides the reporter coding regions, the transgene plasmid contains three major functional elements: the MT enhancer, the *eve* basal promoter and the CaSpeR vector (Fig. 4A). The MT enhancer from the *Drosophila metallothionine* gene interacts with the metal responding factor MTF-1 to drive strong transcriptional activation of either homologous and heterologous genes both *in vivo* and in cultured cells^11,20^. The *eve* basal promoter contains a 42-bp upstream sequence and a canonical TATA box. It exhibits low basal activity but mediates robust activation when combined with a variety of enhancers in cultured cells and transgenic *Drosophila*^12,13,21^. The Casper vector contains *Drosophila* transposon P-elements. We have modified the original Casper vector to remove the adult fly selection marker gene *miniwhite*^9,22–24^. The GFP reporter was cloned into various combinations of enhancer, promoter and vector elements and introduced into *Drosophila* S2 cells via transient transfection. We first tested the role of the MT enhancer in either Cu^++^ or density-mediated GFP induction (Fig. 4B–D). Deletion of the MT enhancer abolished or greatly reduced both Cu^++^- and density-mediated GFP induction (Fig. 4B–D). Replacing MT with 2PE, a mesoderm enhancer from the *twist* gene, which is known to be active in the S2 cells, largely restored the reporter induction by high cell density but not by Cu^++^ (Fig. 4C)^25–27^. However, due to the higher basal expression with this enhancer, the fold of induction by high cell density is low (Fig. 4D). This result suggests that the cell density response is not dependent on the MT enhancer, which is known to mediate signals in multiple responses including heavy metal detoxification and hypoxia responses^20,28,29^. Rather, the MT and 2PE enhancers may serve to augment the density response.

We next replaced the *eve* basal promoter with the *Alcohol dehydrogenase (Adh)* or *Heatshock protein 70 (Hsp70)* promoter. The *Adh* and the *Hsp70* basal promoters have been used widely to drive gene expression in both transgenic *Drosophila* and S2 cells^14,30^. Swapping the *eve* promoter with the *Adh* promoter caused a substantial loss in the magnitude of density-mediated induction but only a small reduction in the relative fold of induction due to a reduced basal transcription level. Replacing the *eve* basal promoter by the *Hsp70* promoter also resulted in a higher background level in density-mediated induction. Taken together, although the *Adh* and *hsp70* promoters both respond to cell density cues, the *eve* promoter provides the most robust response and more suited for diverse applications.

We further evaluated the contribution of the transgene vector by switching the CaSpeR vector with the pTOPO vector (Invitrogen). This dramatically reduced GFP responses to both density and metal inducing cues, although the fold of induction remained comparable to CaSpeR-based combinations (Fig. 4B–D). This result suggests that sequences within the CaSpeR vector may mediate signals from high cell density. Taken together, the transgene dissection analysis indicates that multiple elements in the original MG transgene collaborate to maximize the reporter response to high cell density. Although none of the elements is essential for the density response, the CA-MT-eve combination appears to optimize the reporter induction by high cell density possibly in a synergistic fashion.

### Reporter mRNA induction is contributed in part by hypoxia through the MT enhancer

**Figure 5:**
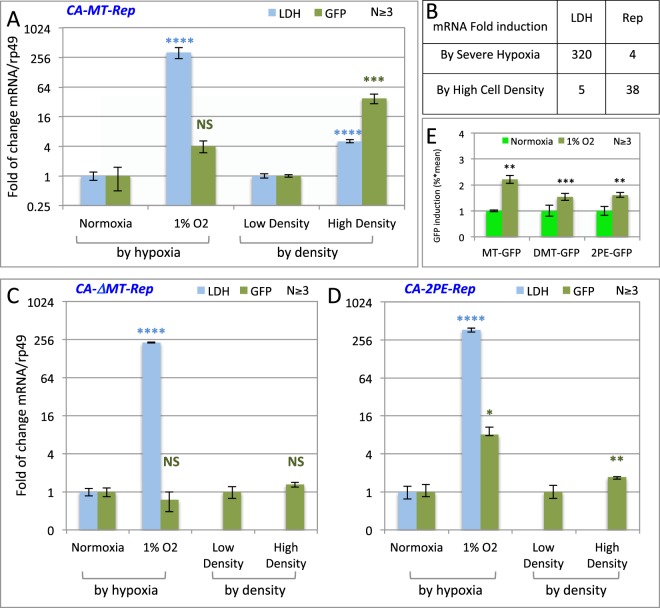
Hypoxia contributes to reporter mRNA induction but not protein induction. (**A**) LDH (blue) and reporter FP (green) mRNA induction by hypoxia (left) and by high cell density (right) in MR transfected cells. The LDH/rp49 or RFP/rp49 mRNA ratio in low-density non-hypoxic cells is used as 1 to calculate fold of induction. (**B**) Summary of relative fold of induction of Reporter FP mRNA by severe (1% O_2_) hypoxia and by high (1.6 × 10^7^/mL) cell density. (**C**) FACS quantitation of RFP fluorescence level under severe hypoxia (1% O_2_). RFP level in low-density, non-hypoxic cells is used as 1 to calculate fold of induction. (**D**) LDH (blue) and reporter FP (green) mRNA induction by hypoxia (left) and by high cell density (right) in ΔMT transfected cells. The LDH/rp49 or GFP/rp49 mRNA ratio in low-density non-hypoxic cells is used as 1 to calculate fold of induction. (**E**) LDH (blue) and GFP (green) mRNA induction by hypoxia (left) and by high cell density (right) in 2PE transfected cells. N indicates the number of biological replicates. The P-values for the difference between the uninduced and induced conditions are marked above the induced data bars.

The role of the MT enhancer in density-driven reporter activation is intriguing. It has been reported that oxygen partial pressure in the pericellular space can be strongly affected by cell density and media height^31–34^. As a result, extreme hypoxia condition may exist in pericellular space under high cell density. Importantly, hypoxia has been shown to activate the *metallothionein* gene through the cooperative binding of MTF-1 and HF-1 factors on the MT enhancer^29,35^. To clarify the roles of hypoxia in reporter induction by high cell density, we compared the mRNA induction of GFP or RFP reporters with an *in vivo* hypoxia marker *lactose dehydrogenase (LDH)* under various hypoxic conditions. We first tested the MT-RFP transgene induction using the hypoxia-mimicking drug Deferoxamine Mesylate (DFO, Fig. S3). After a 24-hour treatment of 50, 100, or 200 µM DFO, concentrations known to induce hypoxia in cultured cells, we observed 3–4-fold increases in the *LDH* mRNA level (Fig. S3)^36–38^. The same conditions did not significantly alter the reporter mRNA level (Fig. S3). Next we tested the effect of low oxygen partial pressure using a hypoxia incubator chamber (see methods). After a 24-hour treatment under 1% O_2_ partial pressure, the *LDH* mRNA level increases by over 300 fold (Fig. 5A,B). In comparison, 1% O_2_ partial pressure only induced reporter mRNA ~4-fold in MT-GFP cells. In contrast, high cell density induced GFP by ~38 fold, but only induced LDH by ~4 fold. These results suggest that although hypoxia can weakly induce reporter mRNA, it cannot account for the dramatic increase of reporter transcription at high cell density. Deletion of the MT enhancer further reduced the reporter response to hypoxia and to crowding (ΔMT, Fig. 5C). Replacing MT with 2PE lead to an increase in the hypoxic response and a decrease in crowding response (2PE, Fig. 5D). This result again indicates that hypoxia is not the major contributor of reporter induction at high cell density.

Hypoxia has been known to suppress protein translation^39–42^. This suppression could potentially affect the reporter read out at high cell density where pericellular hypoxia occurs. To examine whether the mild induction by hypoxia at the mRNA level is reflected in reporter fluorescence, we conducted FACS analysis the MG cells under 1% oxygen partial pressure. We found only a 1–2 fold changes in the fluorescence level under hypoxia condition, compared to the strong GFP or RFP induction by high cell density (Fig. 5E). Taken together, our results indicate that hypoxia is not the major signal that activates the reporter expression at high cell density.

### Reporter induction correlates with changes in several growth-related pathways

**Figure 6:**
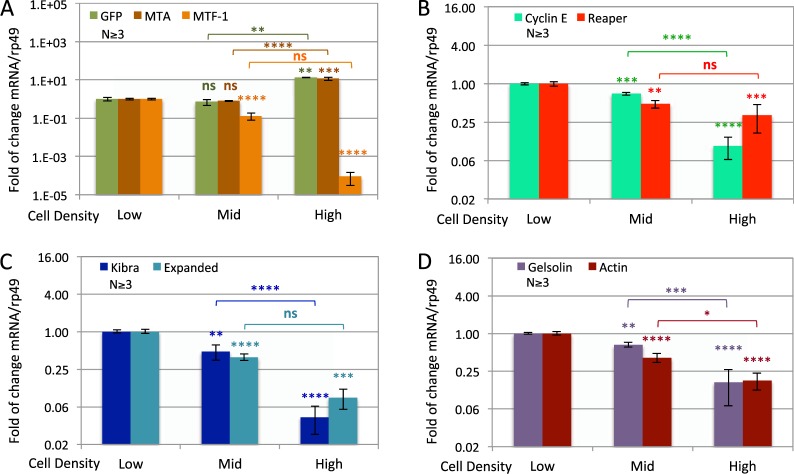
Reporter induction correlates with changes in multiple signaling pathways. (**A**–**D**) Quantitative RT-PCR assessment of GFP and *MTA*-related factors (**A**), cell cycle and apoptosis pathway components (**B**), Hippo pathway components (**C**) and cytoskeletal components (**D**) at low (6 × 10^5^/mL), medium (4 × 10^6^/mL) or high (1.6 × 10^7^/mL) cell density. The mRNA/rp49 mRNA ratio at 4 × 10^6^/mL is used as 1 to calculate fold of induction. N indicates the number of biological replicates. The P-values for the difference between low and medium or high densities are marked above the data bars. Those between medium and high densities are marked with brackets.

Besides hypoxia, many signaling pathways are involved in density sensing and growth control. To begin identifying the signals that activate our reporter expression, we surveyed the concurrent changes in several reporter-related cellular genes and transcription targets of growth-related pathways during the natural crowding process. We first compared the induction of GFP with those of *metallothionein (MTA)*, and *MTF-1*, a transcription factor that binds to the MT enhancers and activates *MTA*^16,17^. We found that both GFP and *MTA* mRNAs increased significantly at high cell density (Fig. 6A). Induction of *MTA* by crowding is not surprising as similar density-dependent activation of *MTA* were reported in HeLa and lymphoma cells, although the changes are more moderate^28^. In addition, *MTA* is also activated by hypoxia via its metal response element^43^. Our results indicate that the transgene reporter is induced in parallel with the *MTA* gene by high cell density, likely through the MT enhancer and contributed by hypoxia. In contrast, the mRNA level of *MTF-1* showed a strong decrease as cells reached medium density and remained low at high density, possibly due to negative feedback.

Next we examined the changes in *Cyclin E (CycE)*, the cyclin that promotes G1-S transition and is overexpressed in many cancer cells^44,45^. We found that the *CycE* mRNA level reduces as cell density increases and possibly enter quiescence at high density. These changes are consistent with the function of *CycE* in growth regulation^46^. We also examined the level of *Drosophila* proapoptotic gene *reaper (rpr)*, a FasC death factor receptor homolog^47–49^. It decreased during the initial phase of growth, and then remained unchanged during the latter phase of growth to high density. In *Drosophila* embryos, *rpr* can be activated by induced hyperplastic growth to restore normal cell density and tissue stoichiometry^50^. However, this was not observed at the high cell density we tested in S2 cells. We next examined *Expanded (Ex)* and *Kibra*, two proteins involved in the contact inhibition of cell proliferation. *Ex* and *Kibra* can signal to the Hippo pathway, leading to the inactivation of *Yorkie (Yki)* and the down regulation of growth and proliferation^51–54^. Both *Ex* and *Kibra* genes are also targets of *Yorkie* activation and therefore are expected to down-regulate at high cell density. This is indeed what we observed – both *Ex* and *Kibra* mRNA showed significant reduction at high cell density (Fig. 6C). Another important pathway related to cell density and proliferation is the cytoskeletal organization. Cancer cells have different cytoskeleton organization^55,56^. Mechanical forces in tissue and cellular environment have been known to act through integrin and actin network to regulate growth^57–59^. The alpha-smooth-muscle Actin (alpha-SMA) and the stress fibers it forms can regulate the Hippo pathway by promoting Yki/YAP nuclear translocation in mammals^52,60–62^. Cytoplasmic beta- and gamma-actin are also known to be down-regulated at high cell density in mouse fibroblasts, often concomitant with F-actin depolymerization^63^. We observed a continued reduction in the mRNA level of *Actin 88F*, the *Drosophila* alpha-SMA, at high cell density (Fig. 6D). We further examined the mRNA level of *gelsolin*, an actin-binding protein that controls the actin filament assembly and disassembly^64–67^. We found it to be also reduced as cell density increases (Fig. 6D). Down-regulation of gelsolin contributes to the disorganized cytoskeleton present in many cancer cells. Our results suggest that both the amount and the organization of the actin network are decreased when S2 cells reach post-confluence density. Taken together, we have examined changes in several cellular pathways related to cell density and growth regulation, hoping to identify transcriptional targets that correlate with the reporter induction both in timing and amplitude. Among the genes we tested, the *MTA* mRNA level responds to cell density in a parallel fashion as the GFP reporter. In comparison, markers of cell cycle regulation, apoptosis, contact inhibition and cytoskeletal organization showed significant but less dramatic changes.

## Discussion

We have characterized a novel reporter system that responds to cell density in transient and stably transformed *Drosophila* S2 cells. We showed that the transgenic reporters are induced in a culture density-dependent and reporter-independent fashion. The rapid and reversible induction of the GFP or RFP transgene is reflected both in the mRNA level and in the mean fluorescence of the cells. These results suggest a quantitative signal(s) that correlates with cell density, and argue against a threshold or all-or-none response. Our results indicate that multiple components within the transgene DNA respond to the density-dependent signals to possibly synergistically induce the reporter expression. Among the elements we tested, the MT enhancer appears to mediate metal response, hypoxia response and high cell density response. Replacing MT with the 2PE enhancer eliminates the responses from the metal and hypoxia but not density-mediated reporter activation, suggesting that distinct signals contribute to reporter induction via these DNA sequences. We further show that the *eve* promoter and the CaSpeR vector sequences in the transgene also help to optimize the density-induced reporter activation. We found that the reporter induction correlates with changes in several growth-related pathways during density increase. These include pathways components involved in cell cycle regulation, apoptosis, contact inhibition and cytoskeleton organization. However, further work is needed to identify the specific signal(s) that directly trigger the reporter induction. Taken together, our analysis suggests that multiple *cis-* and *trans-* factors intercept with cell density and growth regulation signals, supporting the potential of developing versatile and specific reporters that respond to different regulators of cell proliferation.

### The role of the MT enhancer in cell crowding and hypoxia mediated gene regulation

The *metallothionein (MT)* genes encode small cysteine-rich proteins that chelate divalent metal ions like Zn^++^, Cu^++^ and Cd^++^ ^68–70^. They are found widely in eukaryotic species, often in multiple copies and expressed constitutively in most tissues and organs. They are involved in metal metabolism and detoxification, reactive oxygen species scavenging, stress response and neuronal growth regulation^71,72^. Besides responding to metals, certain isoforms of mammalian MT’s (MT-1 and 2) are induced by glucocorticoids while others, such as MT-3, inhibit neurite outgrowth and neuronal survival, and are deficient in patients with Alzheimer’s disease^72^. Therefore, the MT genes are likely to respond to diverse signaling inputs and perform distinct function in different tissues. The MT enhancer we used in this study is isolated from the regulatory region of the *Drosophila MTA* gene^9,11^. *MTA* does not directly correspond to a specific mammalian ortholog but it shares the basic structure and function characteristics of the MT proteins. Previous studies indicate that *MTA* could be induced by several signaling pathways through its enhancer/promoter sequences. Our finding that the MT enhancer responds to Cu^++^, hypoxia, and density-induced signals are also consistent with the previous observations. However, since significant level of reporter activation remains in the absence of the MT enhancer, additional *cis*- and *trans*- signals may also be involved in reporter induction. Further fragmentation or internal deletions may reveal sequences that specifically respond to signal from high cell density.

Hypoxia response is an important mechanism that can drastically alter cell physiology and gene expression profiles. It also affects cell fate decisions including growth and differentiation^73–76^. Our MG reporter responds to low oxygen partial pressure less dramatically than LDH, an *in vivo* hypoxia marker (Fig. 5B,C). But it responds to high cell density more dramatically than LDH, suggesting that although pericellular hypoxia exists under high cell density, it plays a minor role in reporter induction that other density-related signal^31–34^. This is consistent with the observation that the ΔMT transgene, which no longer respond to hypoxia, still respond to high cell density. Importantly, hypoxia is known to suppress protein translation^39–42^. Under severe hypoxia (1% P_O2_) bulk protein synthesis could reduce by over 75% in just two hours^39^. This down regulation is known to be independent of PI3/Akt and HIF-1alpha, but mediated through the repression of mTOR, which acts as an oxygen sensor^77,78^. Consistent with these findings, we observed only a 2-fold increase in the florescent protein level after hypoxia despite the strong increases in the mRNA level. The translation suppression can be alleviated by RNAi knockdown of elF2B-alpha and Tsc-2, two proteins responsible for inhibiting translation under hypoxia, is know to rescue GFP protein expression under hypoxia^39^.

### Developing a cell-based screening platform for regulators of cell density signaling

Cell culture system provides powerful advantages that complement *in vivo* studies. The homogeneity of the cell populations allows consistent and uniform response, thus permitting sensitive and quantitative assessment both in biochemical and cell biological behaviors. The cell-based assays are amenable to chemical and molecular genetic treatment such as drug and small chemical screens, genome-wide RNAi and CRISPR-based screens for identifying genes and pathways regulating a variety of cellular function^79–83^. Transgene reporters can integrate into the genome both via the classic non-homologous insertion of long tandem arrays or through transposase-mediated single copy insertion in different cell clones^84,85^. As a result, these clones may response with different intensity and timing to various signaling events. In particular, the density reporter we reported here provide a foundation for screens for signaling components and regulators of cell cycle progression, apoptosis, contact inhibition of proliferation and cell migration, as well as cytoskeletal organization. The system could be customization at the level of transgene DNA composition, which allows selective response to cell-density and/or hypoxia differentially. Combination of reporter lines containing different transgenes allows multiplex screening for different density-responsive targets. RNAi knock down of elF2B-alpha and Tsc-2 may allow use in hypoxia -related screen. Additional reporters, such as luciferase, could be used for more quantitative readout. The system could also be tested in other cells. In addition to the S2 cells, we have also observed similar reporter response in the *Drosophila* Kc cells (data not shown). Both of these two cells lines are presumed to be mesoderm-derived macrophage-like cells. Although regulation of growth and proliferation occurs in all cell types, different signaling components may be employed to accomplish such regulation. Therefore the assay platform could be tested in additional tissue types to optimize searches for diverse growth-related cell behaviors such as contact inhibition.

## Materials and Methods

### S2 cell culture and transfection

*Drosophila* Schneider’s Line 2 (S2) cells were maintained in HyQ SFX-Insect serum-free medium (HyClone) at 25 °C. Cells were sub-cultured every 6 days. The DNAs used for transfection were prepared using the Qiagen Plasmid Mini Kit. For transfection S2 cells were sub-cultured 3–5 days before transfection, 5 × 10^5^ cells in 1 ml medium were aliquoted into each well of a 12-well plate. After cells had attached to the bottom of the well, they were gently washed once with 1 ml of fresh medium and soaked in 0.5 ml of transfection cocktail (1 μg of assay construct and 2.5 μl of Cellfectin reagent in 0.5 ml medium). For stable transfection, pTurbo plasmid containing the P-element transposase was mixed with the assay construction at a ratio of 1 to 10. The transfection cocktail was replaced with fresh medium after 5 hour of incubation. Cells were normally induced with 1 mM CuSO_4_ 24 hours after transfection. For polyclonal stably transfected cells, transgenic cells were passaged and frequently FACS enriched for eight months until population reach >95% transgenic.

### Construction of DNA plasmids used in S2 cell reporter expression

The pCA-MT-eb-GFP (MG) and pCA-MT-eb-RFP (MR) plasmids were described previously^9,10^. To generate the pCA-Delta-GFP plasmid, the MT element was removed through an EcoRI digestion and the resulting vector religated. To generate the pCA-2PE-GFP plasmid, the MG plasmid was digested with Bam H1 to remove the MT-*eve* promoter, and ligated to a Bam HI fragment containing 2PE-*eve* promoter fragment. For Adh and Hsp70 promoter swap plasmids, the 1.6-kb *Hsp70* promoter and the 1.4-kb *Adh* promoter was PCR cloned and excited out from the pTOPO vector as an EcoR I-BamH I fragment and ligated MT and GFP region (see Supplemental Table 1 for PCR primers). For pTOPO (Invitrogen Cat# 451641) based constructs, the enhancer-promoter-reporter region was removed from CaSpeR-GFP constructs using Hind III and Pst I sites and ligated into the pTOPO vector (Invitrogen Cat# 451641).

### Reporter induction and quantitation

For Cu^++^ induction, CuSO4 is added to the cell media to a final concentration of 1 mM 24 hours prior to FACS analysis or mRNA preparation for qRT-PCR analysis. For crowding induction, cells were cultured for 5–8 days to reach 1.6 × 10^7/mL prior to FACS analysis or mRNA preparation for qRT-PCR analysis. Epifluorescence microscopy and flow cytometry analysis were done 24 hours after induction. Cell images were taken with a digital camera attached to a Zeiss Axioplan 2 fluorescence microscope. FACS was performed using a FACSCalibur flow cytometer (Becton Dickinson Immunocytometry Systems) on 2.5–5 × 10^4^ cells each sample. For each sample, three or more biological replicates were performed. Data analysis was done using the FlowJo software. The fold of induction is calculated as (frequency × mean fluorescence)_induced_/(frequency × mean fluorescence)_uninduced_. For all bargraphs, the number of biological replicates (N) is indicted and the error bar represents standard error of the mean (SEM). P-values for the mean are calculated using the Student’s t-test or ordinary one-way ANOVA multiple comparison in GraphPad Prism or JMP applications. The significance of the differences between means at 95% confidence interval is indicated as follow: four asterisks for P value smaller than 0.0001, three asterisks for P value greater than 0.0001 but smaller than 0.001, two asterisks for P value greater than 0.001 but smaller than 0.01, one asterisks for P value greater than 0.01 but smaller than 0.05, and ns (not significant) for P value greater than 0.05.

### RNA preparation, cDNA preparation, RT-PCR and qRT-PCR

15569018) following the protocol therein. Multiplex RT-PCR reactions using gene-specific primers (see Supplemental Table 1 see primer sequences) were performed using isolated RNAs as templates. Gel electrophoresis was performed on a 2% agarose gel and the specific bands of the product was quantitated using a BioRad gel imager. For the rest of the manuscript, mRNA quantitation was performed using qRT-PCR. Briefly, cDNA library was generated using oligo-d(T) primer and the Superscript III reverse transcriptase Kit (Invitrogen). Quantitative PCR (qPCR) analysis was done to quantify gene expression using SYBR Green Supermix (Bio-Rad). For each sample, at least three biological replicates and two qPCR replicates for each biological replicates were performed. Sequence information for primers used in DNA cloning and RT-PCR is in Supplemental Table 1.

## Supplementary information

Supplemental Figures S1–3 and Table 1

## Data Availability

The authors confirm that the data supporting the findings of this study are available within the article and/or its supplemental materials.
