# Supplementary material for: A Cell Density-Dependent Reporter in the Drosophila S2 Cells
Source: Sci Rep. 2019 Aug 14;9:11868. doi: 10.1038/s41598-019-47652-0 (PMC6694118; doi:10.1038/s41598-019-47652-0)
Supplement: Supplementary file 1 — Supplemental Figures S1–3 and Table 1 [file 41598_2019_47652_MOESM1_ESM.pdf]

A Cell Density-Dependent Reporter in the *Drosophila* S2 Cells

Matthew L Romine[&], Mo Li[&#], Kevin Jiayang Liu, Sapna K Patel, Julie G. Nelson, Ping Shen and Haini N. Cai*

Department of Cellular Biology, University of Georgia, Athens GA, 30602, USA

Keywords: cell culture density, reporter gene, proliferation, hypoxia, Drosophila S2 cells

*: Author to whom correspondence should be addressed. Email: hcai@uga.edu

[&]: These authors contributed equally to the study

[#]: Current Address:

Biological and Environmental Science and Engineering Division,

4700 king Abdullah University of Science and Technology

Thuwal 23955-6900

Saudi Arabia

Supplemental Information

Figure S1: Crowding induction of the RFP reporter gene in S2 cells.

A. Schematic of the pCA-MT-eve-RFP (MR) transgene. Transgene components are as shown as in Figure 1 except the RFP coding region is shown in red. **B-C.** Epifluorescence microscopy images of MR cells at low ($5x10^5$/mL, B) and high ($1.6x10^7$/mL, C) culture density. **D.** Fold of RFP induction by high cell density. Left bar, RFP level in low-density (5x10^5/mL) S2 cells in the absence of $CuSO_4$ is used as a control (=1). Right bar, Fold of RFP induction at high density ($1.6x10^7$/mL) over control. N indicates the number of biological replicates. The P-values for the difference between the RFP means of uninduced and induced conditions is marked above the induced data bar.

Figure S2: Crowding induction of reporters from stably integrated transgenes.

A-B. FACS histogram of MG stable cells at low (5x10^5/mL, A) or high (1.6x10^7/mL, B) culture density. X-axis: log scale of GFP level; Y-axis: cells number at indicated GFP level. Horizontal bar: GFP positive gate with fluorescence level above $2.5x10^3$. C. Quantitation of crowding induction of GFP by FACS. The GFP level in low-density cells is used as 1 to calculate fold of induction. D. Quantitation of crowding induction of GFP mRNA by qRT-PCR using rp49 as a control (see methods for details). The GFP/rp49 mRNA ratio in low-density cells is used as 1 to calculate fold of induction. E-H. FACS histogram of MR stable cells at low (5x10^5/mL, E) or high (1.6x10^7/mL, F) culture density. X-axis: log scale of RFP level; Y-axis: cells number at indicated RFP level. Horizontal bar: RFP positive gate with fluorescence level above $2.5x10^3$. G. Quantitation of crowding induction of RFP by FACS. The RFP level is calculated as the percentage of the RFP positive cells multiplied by the mean RFP intensity of these cells. This level in low-density cells is used as 1 to calculate fold of induction. H. Quantitation of crowding induction of RFP mRNA by qRT-PCR using rp49 as a control (see methods for details). The

RFP/rp49 mRNA ratio in low-density cells is used as 1 to calculate fold of induction. N indicates the number of biological replicates. The P-values for the difference between the uninduced and induced conditions are marked above the induced data bars.

Figure S3. RFP reporter is not induced by up to 200 mM DFO. LDH (blue) and GFP (green) mRNA induction by increasing concentration of DFO in MG transfected cells. The LDH/rp49 or GFP/rp49 mRNA ratio in low-density, 0 μM DFO cells is used as 1 to calculate fold of induction. The P-values are for pair-wise comparisons with the no DFO control. N indicates the number of biological replicates. The P-values for the difference between the uninduced and induced conditions are marked above the induced data bars.

Figure S1

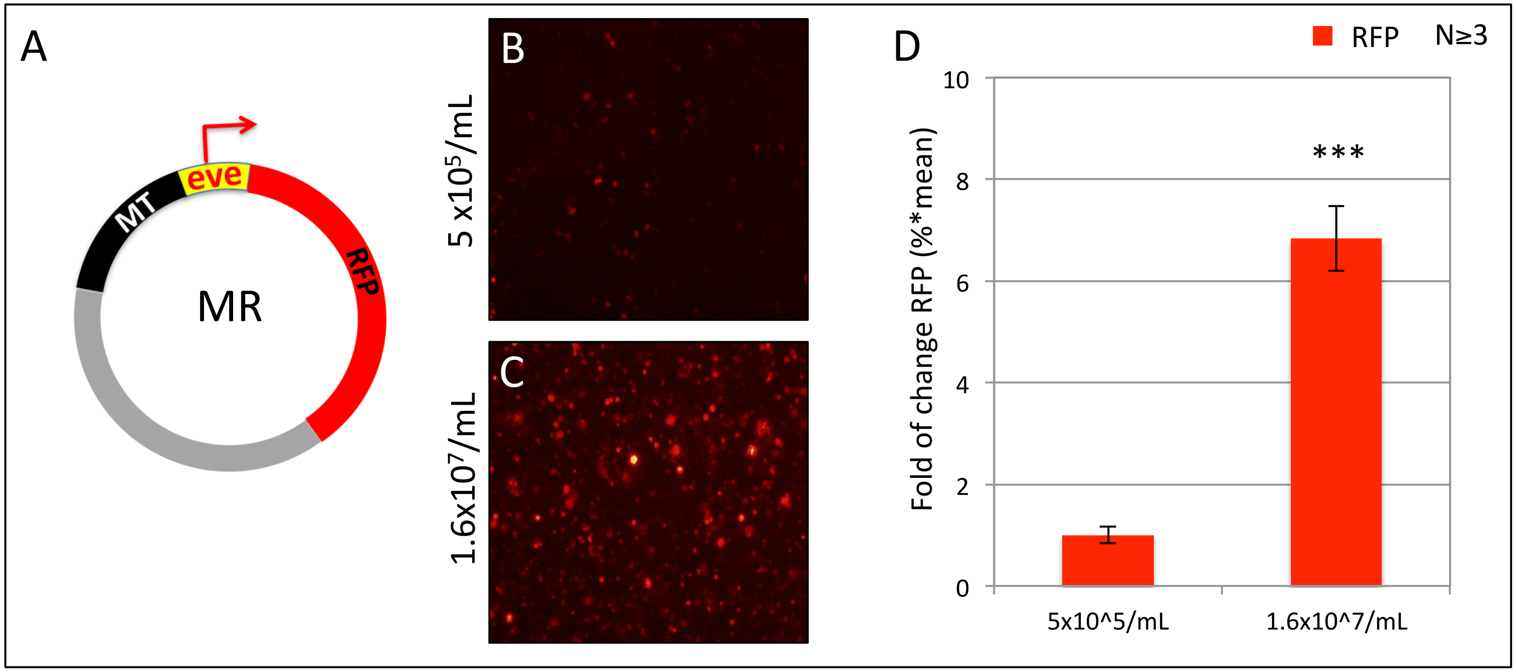

A
MT
eve
RFP
MR
B
5 x10^5/mL
C
1.6x10^7/mL
D
RFP
N≥3
Fold of change RFP (%*mean)
10
8
6
4
2
0
***
5x10^5/mL
1.6x10^7/mL

Figure S2

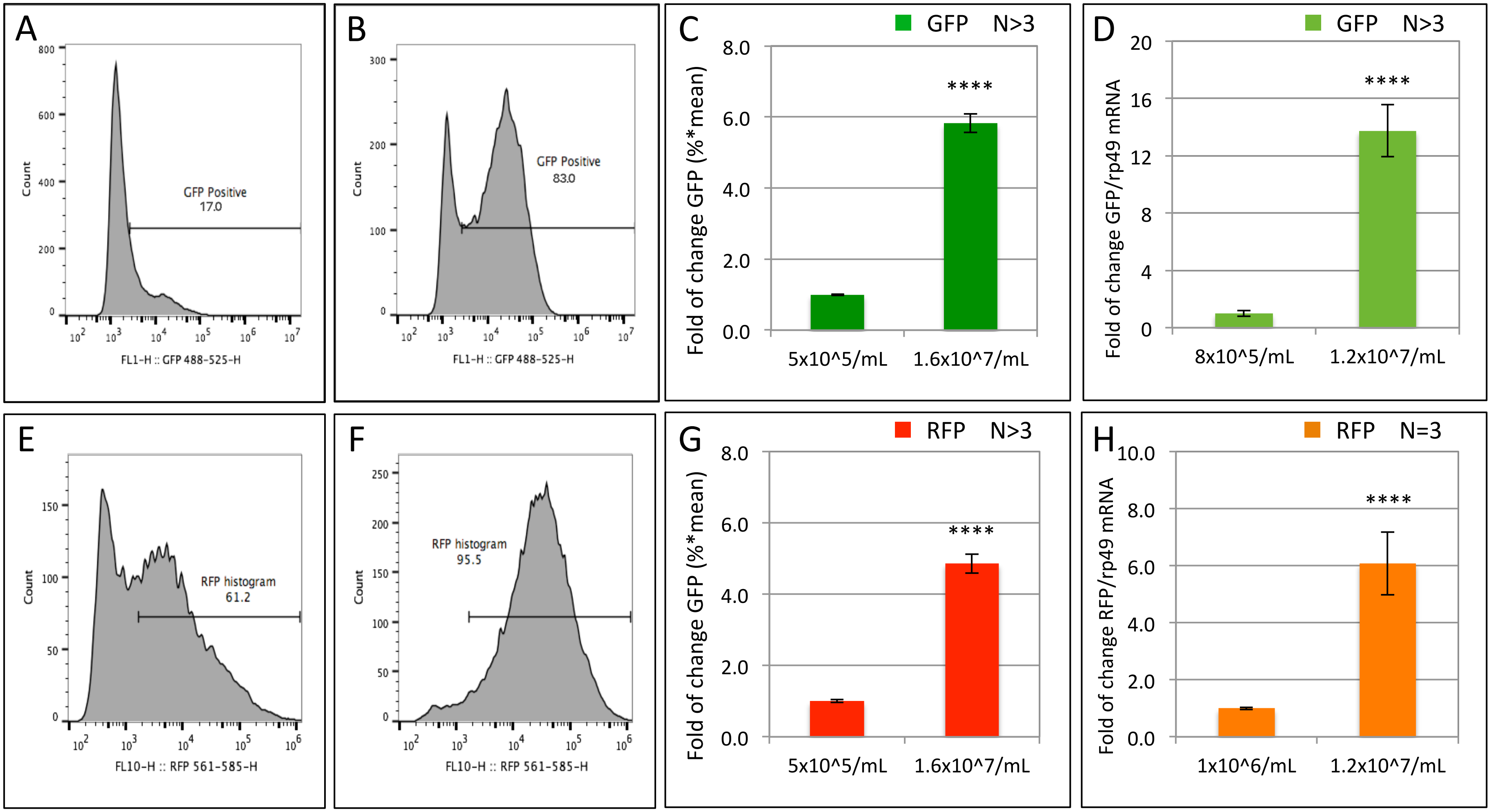

A
Count
GFP Positive
17.0
FL1-H :: GFP 488-525-H
B
Count
GFP Positive
83.0
FL1-H :: GFP 488-525-H
C
GFP N>3
Fold of change GFP (%*mean)
****
5x10^5/mL
1.6x10^7/mL
D
GFP N>3
Fold of change GFP/rp49 mRNA
****
8x10^5/mL
1.2x10^7/mL
E
Count
RFP histogram
61.2
FL10-H :: RFP 561-585-H
F
Count
RFP histogram
95.5
FL10-H :: RFP 561-585-H
G
RFP N>3
Fold of change GFP (%*mean)
****
5x10^5/mL
1.6x10^7/mL
H
RFP N=3
Fold of change RFP/rp49 mRNA
****
1x10^6/mL
1.2x10^7/mL

Figure S3

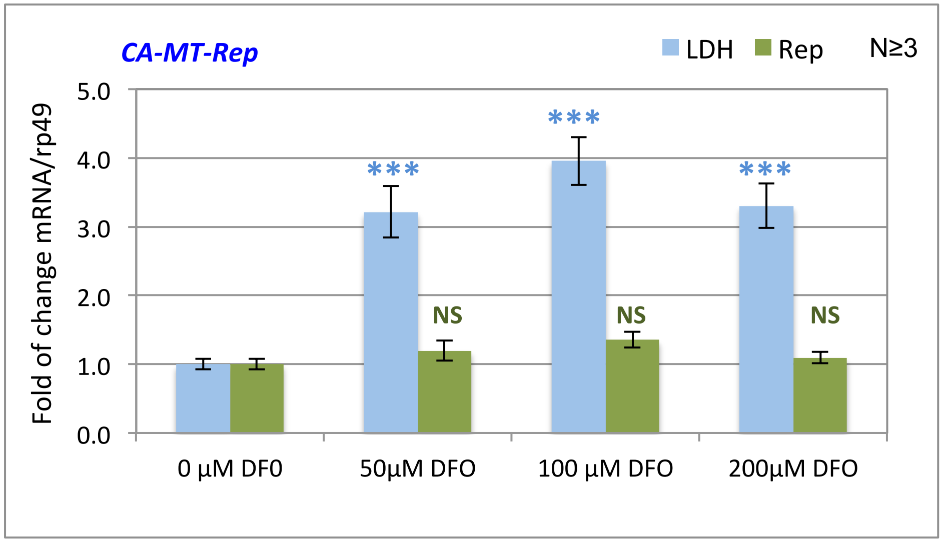

CA-MT-Rep
LDH
Rep
N≥3
Fold of change mRNA/rp49
5.0
4.0
3.0
2.0
1.0
0.0
***
***
***
NS
NS
NS
0 µM DF0
50µM DFO
100 µM DFO
200µM DFO

Supplemental Table 1

Primer sequence for promoter element and RT-PCR

Gene	Forward primer 5'-3'	Reverse primer 5'-3'
ML hsp70 promoter 1.6k	GCGGCCGCGAGAGCTTTGCAGACT	GGATCCGTTTAGCTTGTTCAGCTG
ML Adh promoter 1.4k	GCGGCCGCGATTGATTCTACGCTG	GGATCCTGACTTCTTTTTTGCTTTAG
GFP RT	TGACCCTGAAGTTCATCTGCACCA	TTGATGCCGTTCTTCTGCTTGTCG
RFP RT	ACTACTTGAAGCTGTCCTTCCC	CCCATGGTCTTCTTCTGCATTAC 3'FOR RFP
rp49 RT	CGATATGCTAAGCTGTCGCAC	GGTTCTGCATGAGCAGGAC
MT-A RT	CAACTCAATCAAGATGCCTTGCC	AGCGCCTCTACTCCAGATC
MTF-1 RT	ATTCAACACGCGCTACAGATTG	TGAACTCCTCTTCCTCTTGCT
Cyclin E RT	GCAGCGATTCAAACGAGCTG	GTGAGCTACGTATGCTGAGC
Reaper RT	CAACAATGGCAGTGGCATTC	TCCTCATTGCGATGGCTTG
Merlin RT	CAGGACCTCCACATCAACAAA	CTCTTCGGAACGCCGTTC
Kibra RT	GCCAGTCGAAGCAGTCTGTG	CGACTTGTGCACCTTGAGC
Actin 88F RT	TCTGGACTTCGAGCAGGAGAT	GACAGCACAGAGTTGGCATACA
Gelsolin RT	GAGCTATTTCAAAAACGGCATTCG	TAGCTTCTCAACGCGCTTG
